# Response Mechanism of Polymeric Liquid Junction-Free Reference Electrodes Based on Organic Electrolytes

**DOI:** 10.3390/membranes13010118

**Published:** 2023-01-16

**Authors:** Andrey V. Kalinichev, Nadezhda V. Pokhvishcheva, Maria A. Peshkova

**Affiliations:** Institute of Chemistry, Saint Petersburg State University, 26 Universitetskii Prospect, 198504 Saint Petersburg, Russia

**Keywords:** reference electrodes, polymeric membranes, liquid junction, phase-boundary potential, lipophilic electrolytes

## Abstract

To achieve a transition from conventional liquid-junction reference electrodes (LJF REs) to their all-solid-state alternatives, organic electrolytes are often introduced into the polymeric electrode membranes. In this article, we implement a theoretical approach to the explanation and quantification of the boundary potential stabilization phenomenon for the electrodes modified with organic electrolytes (Q^+^B^−^). For the first time, stabilization of the phase boundary potential due to the partition of lipophilic ions of the Q^+^B^−^ electrolyte between the polymeric and aqueous phases is numerically simulated to predict the LJF electrodes behavior. The impact of the hydrophilic electrolyte on the potential stabilization is demonstrated both numerically and experimentally. The developed model predicted that the small additions of a traditional ion-exchanger enhance performance of the Q^+^B^−^-based reference electrodes. For some specific cases, the optimal concentrations of Q^+^B^−^ and ion-exchanger in the polymeric phase are suggested to provide stable electrode potential in a broad range of aqueous electrolyte concentrations. In addition, the efficiency of the stabilization was shown to be dependent on the overall Q^+^B^−^ load in the polymeric membrane rather than on the closeness of the partition coefficients of the Q^+^ and B^−^ ions; and on the volume of the aqueous phase. The model results are verified experimentally with poly(vinyl chloride) membranes containing ion-exchanger or hydrophilic electrolyte and Q^+^B^−^ in various proportions. A good agreement between the measured electrode response and the theoretical results is observed in a broad range of solution concentrations. In particular, the cationic function of membranes containing KTpClPB is suppressed, and the electrodes begin to behave as REs starting from 50–60 mol. % of ETH500 electrolyte added to the membrane, relative to the total amount of salt.

## 1. Introduction

The role of all-solid-state potentiometric devices in modern electrochemical analysis is hard to overestimate; in particular, there has been a lot of effort to develop robust and miniaturize all-solid-state reference electrodes (SS-REs) [1,2]. One of the major requirements for SS-REs fabrication is solidification of the conventional liquid junction. For this purpose, various organic electrolytes (Q^+^B^−^) are introduced into the composition of the polymeric electrode membrane: moderately lipophilic tetrabutylammonium tetrabutylborate (TBATBB) [3,4], highly lipophilic tetradodecylammonium tetrakis(4-chlorophenyl)borate (ETH500) [5,6] and a number of ionic liquids [7,8,9]. It is generally accepted [10,11] that the partition of the corresponding organic ions of the Q^+^B^−^ electrolyte between the aqueous and polymeric phases imposes a stable potential at the phase boundary; further, it was experimentally demonstrated in Kakiuchi’s [12,13,14] and Bobacka’s [15,16] groups. Although this basic concept has been utilized for over a decade, there are currently no quantitative tools to control and predict the properties of potential reference electrodes, since a quantitative relationship between the phase boundary potential and the composition of the membranes of REs has not been established thus far. Optimization of chemical nature and concentration of the organic electrolytes and conventional active components is performed empirically by sorting through the available substances and their concentrations in the polymeric phase. Only a few attempts were made to assess the conditions for stabilization of the phase boundary potential at the polymeric membrane/solution interface [7,11]. Vincze and Horvai derived that for this purpose “equipartitioning” electrolytes are required, i.e., electrolytes with strictly equal ionic partition coefficients of the cation and anion leading to a stabilized potential equal to zero [11]. Galiullin et al. proposed a more extended theoretical criterion based on the calculated lipophilicities of the cation and anion of the Q^+^B^−^ electrolyte for *a priori* estimation of its applicability for boundary potential stabilization [7]. However, the requirements for the organic electrolyte concentration in the polymeric phase have not been provided.

In this work, we expand the phase boundary potential model described in [17] and elaborated in [10,12,18] for solving the general problem of electrolyte distribution between two immiscible liquids. Our study is now oriented to the related problem of designing reference electrodes without liquid junction, namely to the quantification and prediction of the mechanism of the boundary potential stabilization for the electrode membranes modified with organic electrolytes. In particular, requirements for the nature of organic electrolyte and its concentration in the polymeric phase are proposed for *a priori* optimization of the electrode membrane composition. In addition, the influence of organic electrolyte concentration on the boundary potential stabilization is studied experimentally with ten various membrane compositions in four different hydrophilic electrolytes.

## 2. Materials and Methods

High molecular weight poly(vinyl chloride) (PVC), plasticizers bis(1-butylphenyl)adipate (BBPA) and 2-nitrophenyl octyl ether (oNPOE), potassium tetrakis(4-chlorophenyl)borate (KT*p*ClPB), tetradodecylammonium tetrakis(4-chlorophenyl)borate (ETH500), tetrabutylammonium tetrabutylborate (TBATBB), tetrahydrofuran (THF) and cyclohexanone (CH) (all Selectophore grade) were from Fluka (Buchs, Switzerland). Analytical grade inorganic salts were from Reakhim (St. Petersburg, Russia). Aqueous solutions were prepared with deionized water (18.2 MOhm∙cm, Milli-Q Reference, Merck KGaA, Darmstadt, Germany).

Two series of the membrane cocktails were prepared as described elsewhere [19] by dissolving appropriate amounts of PVC and plasticizer (mass ratio 1:2) in THF. After that, the salts (KT*p*ClPB and ETH500; or TBATBB and KCl if necessary) were added to the cocktails. To avoid weighing small amounts and ensure high accuracy of the membrane compositions, some of the components were added as appropriate aliquots of stock solutions in CH. To obtain the membranes, the cocktails were stirred with roller mixer (J.P. Spectra Movil-Rod, Barcelona, Spain) for 10 min and then casted on a Petri dish and closed with filter paper to slow down the evaporation of THF. In the case of KCl-containing membranes, the cocktail was sonicated for 5 min at 30 °C prior to casting. After 2 days THF and CH completely evaporated and master membranes were obtained. The thickness of TBATBB-based membranes was about 1.7 mm, with TBATBB content of 12.6 wt.% (Table 1). This composition was chosen based on the data in ref. [15] where it was found optimal for creating LJF REs. The amount of KCl added to composition ***b*** (314 mg, see Table 1) was in a mass ratio of 1:1 to the oNPOE plasticizer. The thickness of the membranes ***I–X*** was 1 mm, and the total amount of the organic electrolytes (KT*p*ClPB and ETH500) in these compositions was kept constant, 10 mmol/kg BBPA; the mole fraction of ETH500 was varied from 0.0 to 0.9 (see Table 1).

The electrodes were prepared by cutting disks with diameter of 12 mm from the master membrane and gluing them to PVC bodies with the outer diameter of 12 mm and the inner diameter of 10 mm. A solution of PVC in CH was used as glue. The electrodes from the series ***I–X*** were filled with 10^−2^ M KCl, NH_4_Cl, CsCl or NaCl and conditioned in the same solution for 2 days. The conditioning protocols of the membranes of series ***a***, ***b*** will be discussed below. The photographs of the fresh and conditioned membranes containing TBATBB and KCl are shown in Figure S1.

The inner reference was Ag/AgCl wire. Potentiometric measurements were performed versus saturated Ag/AgCl electrode with Ecotest-120 potentiometer (Econics, Moscow, Russia) at 23 ± 1 °C.

For data processing, OriginPro 9.0 software (OriginLab Corporation, Northampton, MA, USA) was used. Numerical simulations of the phase boundary potential were performed with Maple 2016 (Maplesoft, Waterloo, ON, Canada).

## 3. Result and Discussion

As a characteristic example, let us consider the following case: hydrophilic 1:1-electrolyte M^+^X^−^ dominates in the solution; polymeric phase contains organic electrolyte Q^+^B^−^ and/or cation exchanger I^+^R^−^.

The proposed model is based on the equality of the electrochemical potentials of the charged species in the two phases (*aq*—aqueous phase, *m*—polymeric phase):

(1) $$\mu_{i}^{aq,o}+RT\ln a_{i}^{aq}+z_{i}F\varphi^{aq}=\mu_{i}^{m,o}+RT\ln a_{i}^{m}+z_{i}F\varphi^{m}$$

were $\mu_{i}^{\alpha ,\;o}$—standard chemical potential of the i-th ion in the α phase, $\varphi^{\alpha }$—Galvani potential of α phase, $a_{i}^{\alpha }$—activity of the i-th ion in the α phase, $z_{i}\;$—charge of the i-th ion. Applying the generally accepted approach, one can approximate activities in the polymeric phase with the respective concentrations ($a_{i}^{m}\equiv C_{i}^{m}$) [20], and the activity coefficients for all ions in all solutions are equal to 1. Let us introduce the following notation: $\left[i\right]≝C_{i}^{m}$ and $a_{i}≝a_{i}^{aq}$.

Thus, the boundary potential can be written as follows:

(2) $$\varphi_{b}=\varphi^{m}-\varphi^{aq}=-\frac{\mu_{i}^{m,o}-\mu_{i}^{aq,o}}{z_{i}F}+\frac{RT}{z_{i}F}\ln\frac{a_{i}}{\left[i\right]}$$

with $\Phi =\exp\left(\frac{\varphi_{b}F}{RT}\right)$:

(3) $$\Phi ={\left(\frac{k_{i}a_{i}}{\left[i\right]}\right)}^{z_{i}\;}$$

where $k_{i}\;$—Eisenman ionic partition coefficient of ion i [21]. Thus, for the partitioning species:

(4) $$\Phi =\frac{k_{M}a_{M}}{\left[M^{+}\right]}=\frac{k_{Q}a_{Q}}{\left[Q^{+}\right]}=\frac{k_{I}a_{I}}{\left[I^{+}\right]}=\frac{\left[X^{-}\right]}{k_{X}a_{X}}=\frac{\left[B^{-}\right]}{k_{B}a_{B}}=\frac{\left[R^{-}\right]}{k_{R}a_{R}}$$

The mass balance is expressed in terms of the total quantity of substances (n) added into each phase [11] with volumes of the aqueous and the polymeric phase be $V_{aq}$ and $V_{m}$, respectively. Then for MX mass balance:

(5) $$n\left(MX\right)=V_{aq}a_{M}+V_{m}\left[M^{+}]=V_{aq}a_{X}+V_{m}[X^{-}\right]$$

Q^+^B^−^ electrolyte mass balance:

(6) $$n\left(QB\right)=V_{aq}a_{Q}+V_{m}\left[Q^{+}\right]=V_{aq}a_{B}+V_{m}\left[B^{-}\right]$$

IR electrolyte mass balance:

(7) $$n\left(IR\right)=V_{aq}a_{I}+V_{m}\left[I^{+}\right]=V_{aq}a_{R}+V_{m}\left[R^{-}\right]$$

Electroneutrality needs to be considered only for one of the phases since by introducing the mass balance equation, the electroneutrality condition for another phase would be automatically fulfilled. Then for the polymeric phase one can write:

(8) $$\left[M^{+}\right]+\left[Q^{+}\right]+\left[I^{+}\right]=\left[X^{-}\right]+\left[B^{-}\right]+\left[R^{-}\right]$$

In computer simulations, unless otherwise indicated, the following fixed parameters close to those found in the literature were used [7,19]: $k_{M}=10^{-6}$,$\;k_{X}=10^{-4};\;k_{Q}=10^{3}$, $k_{B}=10^{2}$; $k_{I}=10^{-5}$, $k_{R}=10^{9}$; $V_{aq}=10^{-3}\;L$, $V_{m}=10^{-5}\;L$.

**Figure 1:**
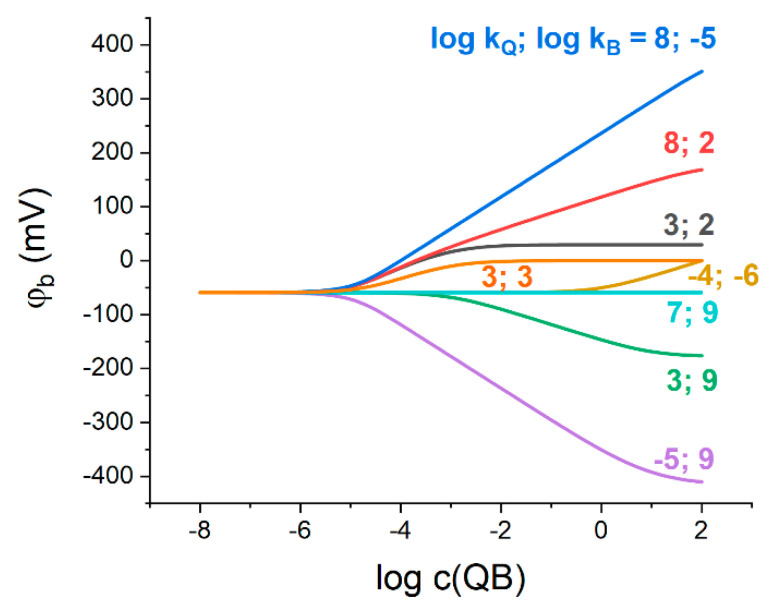
Phase boundary potential simulated at arbitrary Q^+^ and B^−^ ionic partition coefficients (indicated in the plot in log units) vs. Q^+^B^−^ concentration in the membrane phase. $c\left(MX\right)=10^{-3}\;M$. Here and below the concentrations of the membrane species refer to the entire volume of the polymeric phase.

Figure 1 shows the results of simulation of the phase boundary potential, $\varphi_{b}$, as a function of Q^+^B^−^ concentration in the membrane phase with varied ionic partition coefficients of Q^+^ and B^−^. We note that the initial concentration of Q^+^B^−^ is considered without taking into account its leaching to the aqueous phase.

According to Figure 1, in a wide range of Q^+^B^−^ concentrations below ca. 10^−5^ M, the potential does not depend on the ratio of Q^+^ and B^−^ ionic partition coefficients, nor on their absolute values. Thus, in the case of a low concentration of the organic electrolyte in the membrane, the phase boundary potential is determined exclusively by the hydrophilic salt partitioning into the membrane phase.

In order to develop a reference electrode, the phase boundary potential should be maintained constant. The proposed model suggests that the limiting values of $\varphi_{b}$ in Figure 1 depend on the ratios of the partition coefficients of both hydrophilic and lipophilic electrolytes:

(9) $$\underset{c\left(QB\right)\to 0}{\lim}\varphi_{b}=\frac{RT}{2F}\ln\frac{k_{M}}{k_{X}}$$

(10) $$\underset{c\left(QB\right)\to +\infty }{\lim}\varphi_{b}=\frac{RT}{2F}\ln\frac{k_{Q}}{k_{B}}$$

Figure 1 indicates that a necessary condition for the curves to reduce to horizontal lines, which is equivalent to $\varphi_{b}$ to be constant at any concentration of Q^+^B^−^, is that the two limits are equal:

(11) $$\frac{k_{M}}{k_{X}}=\frac{k_{Q}}{k_{B}}$$

An example can be observed for $k_{M}=10^{7},\;k_{X}=10^{9}$ (see turquoise line in Figure 1, note that $k_{M}=10^{-6}$,$\;k_{X}=10^{-4}$, see above) that expands the previously adopted concept of the partitioning electrolyte [11] to the non-zero values of the stabilized boundary potential. The latter also confirms the qualitative conclusion of ref. [7]: the equality of the Q^+^ and B^−^ ionic partition coefficients in Q^+^B^−^ electrolyte is not obligatory for this electrolyte to stabilize the interfacial potential.

**Figure 2:**
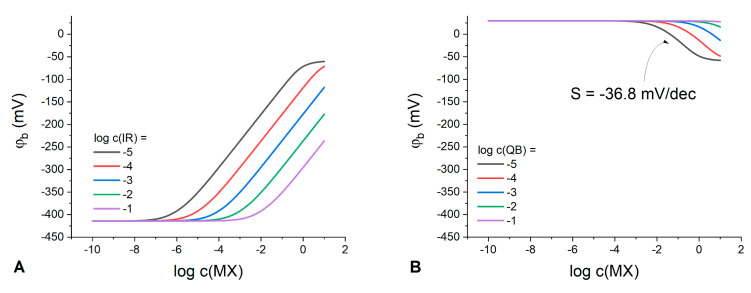
Calculated dependencies of the phase boundary potential on MX concentration in the solution at varied IR (**A**) or Q^+^B^−^ (**B**) content in the polymeric membrane (indicated in the plot). $k_{Q}=10^{3}$, $k_{B}=10^{2}$; $k_{M}=10^{-6}$, $k_{X}=10^{-4}$;$\;k_{I}=10^{-5}$, $k_{R}=10^{9}$.

The dependence of the phase boundary potential on the IR or Q^+^B^−^ electrolyte total concentration at varied MX concentration is shown in Figure 2.

Expectedly, the classic Nernstian behavior of the boundary potential with a slope of 59.1 mV/dec can be observed at any IR concentration in membranes containing solely an ion exchanger (Figure 2A). Interestingly, at any IR concentration in the polymeric phase, there is a domain in the response curve where the boundary potential remains stable. For example, at IR concentration 10^−1^ M the membrane demonstrates stable interfacial potential up to 10^−3^ M MX in the solution. Thus, in principle, any organic electrolyte can be used to stabilize the phase boundary potential with respect to variations of the aqueous electrolyte; the latter being consistent with the results obtained in ref. [10].

**Figure 3:**
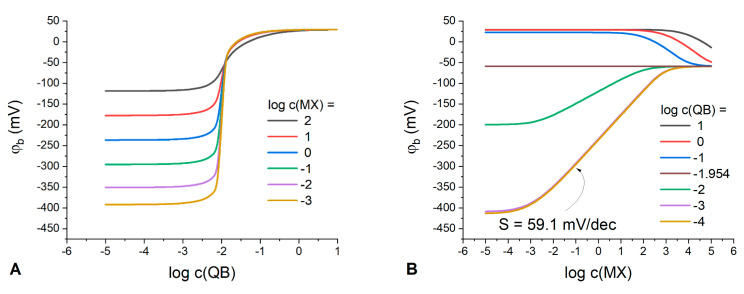
Phase boundary potential simulated: (**A**) vs. Q^+^B^−^ concentration in the polymeric phase at different MX concentrations in the aqueous phase (indicated in the plot); (**B**) vs. MX concentration in the aqueous phase at different Q^+^B^−^ concentration in the polymeric phase (indicated in the plot). $c\left(IR\right)=10^{-2}\;M$; $k_{M}=10^{-6}$,$\;k_{X}=10^{-4}$; $k_{Q}=10^{3}$, $k_{B}=10^{2}$; $k_{I}=10^{-5}$, $k_{R}=10^{9}$.

Meanwhile, for membranes containing only the Q^+^B^−^ electrolyte (Figure 2B), the response to the solution concentration is substantially suppressed and the interfacial potential is stable up to 10^−3^ M of MX the aqueous electrolyte already at a Q^+^B^−^ content as low as 10^−5^ M. The higher the Q^+^B^−^ concentration in the membrane, the wider the range of MX concentrations where the potential remains stable. The membranes with low content of Q^+^B^−^ exhibit a sub-Nernstian response with a slope of −36.8 mV/dec over a relatively narrow concentration range (Figure 2B), with the response becoming anionic as compared to IR-containing membranes. The nature of this response can be explained by the process of anions coextraction due to the absence or deficiency of uncompensated anionic sites and, accordingly, of the cation-exchange capacity. This explanation is confirmed by the species concentration profiles (Figure S2) simulated for the two different values of partition coefficient for the aqueous anion X^−^. One can see that in the case of Q^+^B^−^ electrolyte, the electrode response to MX concentration (Figure 2B) appears due to the anion X^−^ interference caused by the effect of coextraction. Thus, to optimize the RE composition, it appears logical to ensure Donnan exclusion in the polymeric phase by introducing some amount of a cation exchanger (IR) into the membrane phase containing Q^+^B^−^. The phase boundary potential was simulated at various molar ratios of Q^+^B^−^ to IR electrolytes added simultaneously to the membrane (Figure 3).

Figure 3A shows that the combination of Q^+^B^−^ and IR in the polymeric phase leads to a new shape of the dependences $\varphi_{b}-c\left(QB\right)$ as compared to Figure 1, with a drastic potential jump at nearly equimolar IR to Q^+^B^−^ ratio, followed by a plateau, with the location of the latter being independent on MX concentration. One can see from Figure 3 that at c(QB)<c(IR), the phase boundary potential curve demonstrates a Nernstian slope within its linear range (see Figure 3B), whereas at c(QB)>c(IR), a potential stabilization is observed in a wide range of MX concentrations (up to 10 M).

**Figure 4:**
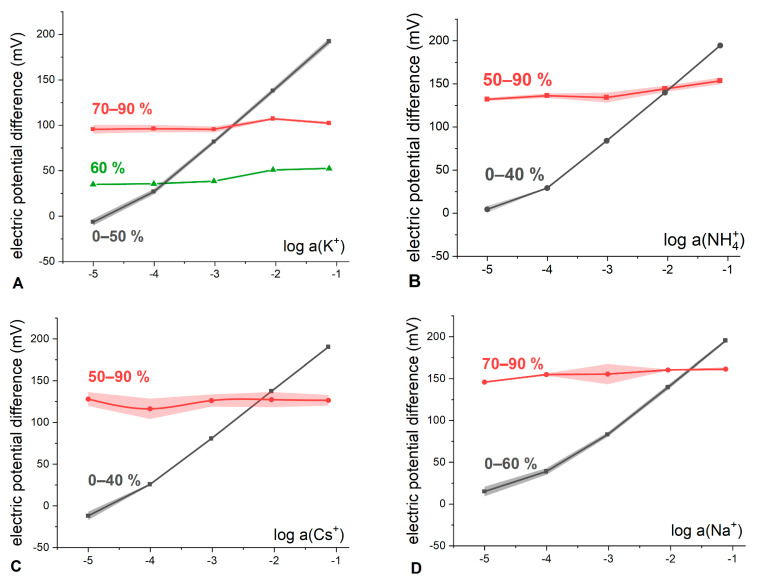
The response of the electrodes containing both KT*p*ClPB and ETH500 in the membrane, to potassium (**A**); ammonium (**B**); cesium (**C**), and sodium (**D**) ionic activity. ETH500 content was varied from 0 to 90 mol. % of the overall salt quantity (indicated in the plots). When possible, for better clarity, the values of E for the membranes with different ETH500 content were averaged and the resulting dispersion of the potential difference is represented as shaded areas.

To qualitatively illustrate a drastic change of the boundary potential curves, the electrode membranes containing both IR (KT*p*ClPB) and Q^+^B^−^ (ETH500) were prepared and their calibrations in KCl, NH_4_Cl, CsCl and NaCl solutions were performed (Figure 4).

Indeed, it can be observed that at sub-stoichiometric ratios of ETH500 to KT*p*ClPB content (black curves in Figure 4) the measured electric potential difference (E) exhibits near-Nernstian dependence on the hydrophilic electrolyte activity with the slopes from 57 to 59 mV/dec (see Table S1) in the solutions of KCl, NH_4_Cl, CsCl and NaCl. At ETH500 to KT*p*ClPB ratios above equimolar (red curves in Figure 4), the cationic function is lost, and the electrode begins to behave as a RE with the slopes as low as 1–5 mV/dec and average potential differences ranging from 100 to 155 mV. The values of E practically do not depend on the further increase in the ETH500 content, as predicted by the developed theoretical formalism.

**Figure 5:**
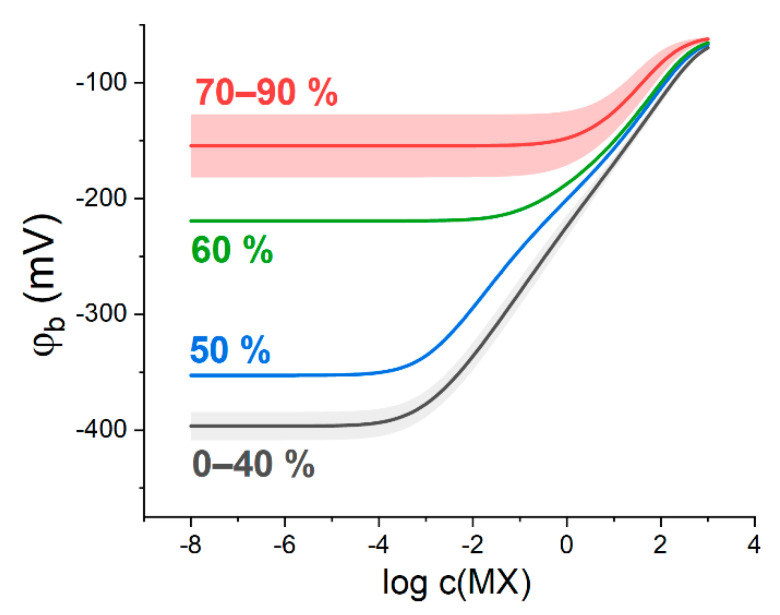
The simulated response curves for the membranes containing both IR and Q^+^B^−^ in molar ratios from 0 to 40% (black curve), 50% (blue curve), 60% (green curve) and from 70 to 90% (red curve). When possible, for better clarity, the values of E for the membranes with different IR content were averaged and the resulting dispersion of the potential difference is represented as shaded areas (see Figure S3 for all the individual curves). $k_{M}=10^{-6}$,$\;k_{X}=10^{-4}$; $k_{Q}=10^{9}$, $k_{B}=10^{5}$; $k_{I}=10^{-5}$; $k_{R}=10^{9}$; $c\left(QB+IR\right)=10^{-2}\;M$.

In parallel with the experiment, the response of membranes of the same composition was simulated at parameter values close to the experimental ones (Figure 5).

Comparison of Figure 4 and Figure 5 suggests a good qualitative correspondence between experimental and simulated behavior of the membranes containing both salts. Experimental results do not contain a domain with cationic response in the most concentrated solutions, as opposed to the simulation. Meanwhile, in the case of KCl solutions (Figure 4A) it was possible to register a response curve (60% of ETH500, green curve in Figure 4A) which demonstrates a transition from the conventional cation-selective response (0 to 40% in Figure 4A) to the RE-like behavior (70 to 90% in Figure 4A). The slope of the response is already negligible (5.2 ± 1.2 mV/dec), but the mean value of potential difference is only 42.5 ± 8.6 mV which is much lower than the respective values for the membranes with higher ETH500 content. This type of “transitional” curves was obtained numerically as well (50 and 60%, blue and green lines in Figure 5).

**Figure 6:**
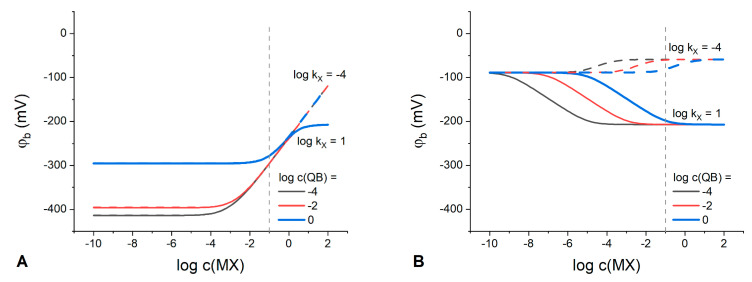
Phase boundary potential simulated vs. MX concentration in the aqueous phase at different Q^+^B^−^ concentrations in the polymeric phase (indicated in the plot) at varied lipophilicity of anion X^−^ (10 and 10^−4^ for solid and dotted lines, respectively): (**A**) $c\left(IR\right)=10^{-2}\;M$; (**B**) no added IR electrolyte. $k_{M}=10^{-6}$; $k_{Q}=10^{6}$, $k_{B}=10^{9}$; $k_{I}=10^{-5}$, $k_{R}=10^{9}$.

The influence of anion X^−^ lipophilicity on the interfacial potential of the membranes containing highly lipophilic organic electrolyte Q^+^B^−^ ($k_{Q}=10^{6}$, $k_{B}=10^{9}$) and cation exchanger IR ($k_{I}=10^{-5}$, $k_{R}=10^{9}$) to MX concentration in the solution is demonstrated in Figure 6A. For comparison, Figure 6B shows anion effect in the response of Q^+^B^−^-containing but IR-free membranes.

Based on the comparison of Figure 6A and 6B, one can observe that the addition of IR to the Q^+^B^−^-containing membranes crucially affects their response patterns with respect to anion interference. In the case of the membranes additionally doped with IR, the lipophilicity of the solution anion has almost no effect on the values of interfacial potential and its stability up to as high as 1 M MX solution (Figure 6A). Moreover, in the presence of the IR electrolyte, the phase boundary potential is effectively stabilized with 1 M Q^+^B^−^ in the membrane up to 10^−1^ M MX solution regardless $k_{X}$ value (blue line in Figure 6A). Oppositely, the behavior of the membranes containing only the Q^+^B^−^ salt strongly depends on the lipophilicity of the solution anion X^−^ (Figure 6B). At the absence of the IR electrolyte, the phase boundary potential is nearly constant up to 10^−1^ M MX at $k_{X}=10^{-4}$, while for $k_{X}=10$ it is stable only up to 10^−5^ M MX, for the same concentration of Q^+^B^−^, 1 M. In addition, the span of the coextraction-induced anionic response is much larger in the case of a more lipophilic solution anion. Thus, to create polymeric REs based on lipophilic salts, it is beneficial to add a cation exchanger to the membrane along with the Q^+^B^−^ but in the concentrations lower than those of organic electrolyte.

**Figure 7:**
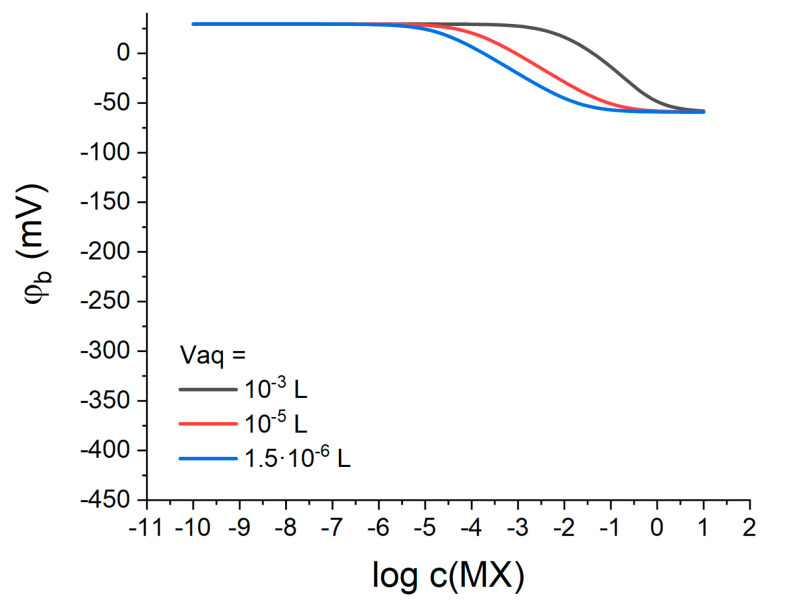
The simulated response of Q^+^B^−^-containing membranes to aqueous electrolyte concentration for various volumes of the aqueous phase (indicated in the plot). Simulation parameters: $c\left(QB\right)=10^{-5}\;M$, $V_{m}=10^{-5}\;L;\;k_{M}=10^{-6}$,$\;k_{X}=10^{-4}$; $k_{Q}=10^{3}$, $k_{B}=10^{2}$.

Following [11], our model also suggests that the response of Q^+^B^−^-containing membranes should depend on the volumes of both polymeric and aqueous phases. To track this dependence, the response was simulated for various volumes of the aqueous phase at otherwise constant parameters (see above). The volume of the membrane phase was $10^{-5}\;L$, which is a typical value for an ion-selective electrode membrane (diameter 8 mm, thickness 0.2 mm). The volume of the aqueous phase was $10^{-3}\;L$, as in all previous simulations; or $10^{-5}\;L$, which is equal to the volume of the polymeric phase; or $1.5\cdot 10^{-6}\;L$, which is close to the volume of an aqueous diffusion layer 30 μm thick [22] for a membrane with a diameter of 8 mm. The simulation results are shown in Figure 7.

One can see from Figure 7 that; indeed, the stability of the interfacial potential depends significantly on the volume of the aqueous phase. A decrease in the solution volume narrows the range of potential stability along the MX concentration axis and shifts it towards more dilute solutions. Compare the blue curve for the volume $1.5\cdot 10^{-6}\;L$ and the black curve for $10^{-3}\;L$: the potential is stable up to $10^{-6}\;M$ MX for a smaller solution volume, and up to $10^{-3}\;M$ in the case of a larger volume. Meanwhile, the range of the anionic response in the first case is wider: ca. 10^−6–^10^−1^ M MX for $1.5\cdot 10^{-6}\;L$ solution volume and from 10^−3^ to 1 M for volume of $10^{-3}\;L$. Thus, in order to optimize the behavior of LJF reference electrodes based on lipophilic salts it is necessary to take into account the volume ratio of the membrane and the solution and ensure a sufficient excess of the latter.

**Figure 8:**
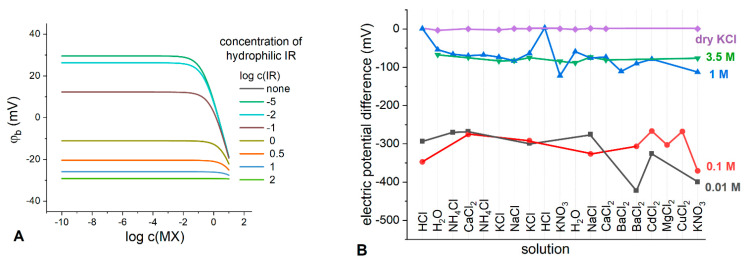
(**A**) Simulated boundary potential for the membranes containing Q^+^B^−^ and hydrophilic electrolyte (IR) added to the membrane in various concentrations (indicated in the plot). c(QB)=0.15 M; $k_{I}=10^{-5}$, $k_{R}=10^{-4};\;k_{Q}=10^{3}$, $k_{B}=10^{2};\;V_{m}=10^{-5}\;L,\;V_{aq}=1.5\cdot 10^{-6}\;L;\;k_{M}=10^{-6}$,$\;k_{X}=10^{-4}$. (**B**) Experimental response of the electrodes with lipophilic electrolyte TBATBB and salt KCl in the membrane, in various electrolyte solutions (indicated in the plot). Soaking solutions: 0.01 M KCl (black curve), 0.1 M KCl (red curve), 1 M (blue curve), 3.5 M (green curve); violet curve: KCl was added as dry salt into the membrane.

It is generally accepted that the addition of the salt bridge electrolyte, e.g., KCl, to the Q^+^B^−^-based membranes enhance the characteristics of the resulting LJF reference electrodes. It was experimentally confirmed in [15,23,24], but has never been discussed theoretically. We performed numerical simulation of the boundary potential according to the proposed model, taking into account the partition of a hydrophilic electrolyte between the polymeric phase and the diffusion layer of the aqueous phase, see Figure 8A. Now, IR is a hydrophilic electrolyte added to the membrane, MX is a solution electrolyte.

As can be seen from Figure 8A, even at relatively high concentrations of lipophilic electrolyte Q^+^B^−^, the addition of a hydrophilic electrolyte to the membrane affects the stability of the phase boundary potential: the higher the concentration of hydrophilic IR, the wider is the range of potential stability. Apparently, partition of the hydrophilic ions contributes to the overall effect of potential stabilization.

To experimentally track the effect of hydrophilic electrolyte content in the membranes containing Q^+^B^−^, we prepared the membranes containing TBATBB salt which was found earlier to be optimal for developing LJF REs [15]. The electrodes were prepared, and their TBATBB-based membranes were loaded with KCl in different ways: by soaking the membranes in the solution of KCl with concentrations of 0.01 M, 0.1 M, 1 M and 3.5 M (saturated), and, finally, by introducing dry KCl into the membrane during its fabrication. The electric potential difference was measured against saturated Ag/AgCl electrode in the solutions of different nature and concentration. The results are shown in Figure 8B. As expected, the membranes soaked in the least concentrated 0.01 M KCl solution showed the least stable potential in the entire range of the studied electrolytes with an average potential difference of −319.5 ± 60.0 mV. Increasing the concentration of the soaking solution leads to more stable potential differences, meanwhile their average values increase: −306.1 ± 36.2 mV, −70.3 ± 33.3 mV, and −78.6 ± 6.5 mV for 0.1 M, 1 M and 3.5 M (saturated) soaking solutions, respectively. The membranes containing dry KCl along with TBATBB demonstrated an extremely stable potential of 0 ± 1.6 mV for the entire range of the studied electrolytes. This finding is consistent with the results of the simulation performed in this work, as well as with the experimental data obtained earlier [15,23,24].

## 4. Conclusions

In this work, we expanded the phase boundary model by considering the quantitative and qualitative composition of both aqueous and polymeric phases in order to describe the formation of the phase boundary potential in liquid junction-free reference electrodes based on lipophilic salts. To summarize, the following observations can be utilized to optimize the behavior of the salt-based LJF reference electrodes.

It was demonstrated that, generally, the phase boundary potential can be stabilized by loading the polymeric membrane with any organic electrolyte (Q^+^B^−^), including a conventional ion-exchanger, although for the latter, the stabilization of potential cannot be achieved at practically realistic concentrations. However, it was shown that small quantities of ion-exchanger added to the membrane along with the Q^+^B^−^ electrolyte, suppress the coextraction-mediated electrode response thereby improving the behavior of the resulting reference electrodes. The closeness of the partition coefficients of the Q^+^B^−^ ions was shown to not play a key role in potential stabilization. Increasing the content of Q^+^B^−^ in the polymeric phase improves the stability of the boundary potential. A sufficient excess of the solution volume over the membrane should be provided in order to achieve higher stability of the boundary potential. Finally, the beneficial effect of doping Q^+^B^−^-based membranes with a hydrophilic electrolyte was simulated numerically and confirmed experimentally.

The obtained experimental results are qualitatively consistent with theoretical conclusions. Quantitative agreement requires additional experimental data on the water-PVC partition coefficients of the species under consideration, which may be the subject of a separate study. Nevertheless, the reported study opens an avenue for *a priori* optimization of the electrode membrane composition to enhance the performance of the reference electrodes without liquid junction.

## Figures and Tables

**Table 1:** Composition of the prepared electrode membranes.

Membrane	*a*	*b*	*I*	*II*	*III*	*IV*	*V*	*VI*	*VII*	*VIII*	*IX*	*X*
plasticizer	oNPOE		BBPA									
KT*p*ClPB, mmol/kg BBPA	-		10	9	8	7	6	5	4	3	2	1
ETH500, mmol/kg BBPA	-		0	1	2	3	4	5	6	7	8	9
ETH500, mol.%	-		0	10	20	30	40	50	60	70	80	90
TBATBB, wt.%	12.6		-									
TBATBB, mmol/kg oNPOE	450											
dry KCl, mass ratio to plasticizer	-	1:1	-									
dry KCl, wt.%		36.8										

## Data Availability

The data presented in this study are available on request from the corresponding author. The data are not publicly available due to privacy reasons.

## Supplementary Materials

The following are available online at https://www.mdpi.com/article/10.3390/membranes13010118/s1, Figure S1: photographs of the fresh and conditioned membranes containing TBATBB and KCl, Figure S2: calculated dependences of the phase boundary potential on the Q^+^B^−^ content in the polymeric phase at varied MX concentration in the aqueous phase for two different partition coefficients of the aqueous anion X^−^ and the respective concentration profiles in the polymeric phase, Figure S3: the simulated response curves for the membranes containing both IR and Q^+^B^−^ in molar ratios from 10 to 90%, Table S1: experimental characteristics of the response of the electrodes containing both KT*p*ClPB and ETH500 electrolytes in the membrane.
