# Supplementary material for: Response Mechanism of Polymeric Liquid Junction-Free Reference Electrodes Based on Organic Electrolytes
Source: Membranes (Basel). 2023 Jan 16;13(1):118. doi: 10.3390/membranes13010118 (PMC9864460; doi:10.3390/membranes13010118)
Supplement: Supplementary file 1 [file membranes-13-00118-s001.zip › membranes-2152458-supplementary.pdf]

membranes

Supplementary material

Response Mechanism of Polymeric Liquid Junction-Free Reference Electrodes Based on Organic Electrolytes

Andrey V. Kalinichev *, Nadezhda V. Pokhvishcheva and Maria A. Peshkova

Institute of Chemistry, Saint Petersburg State University, 26 Universitetskii prospect, 198504 Saint Petersburg, Russia
* Correspondence: andre.kalinichev@gmail.com

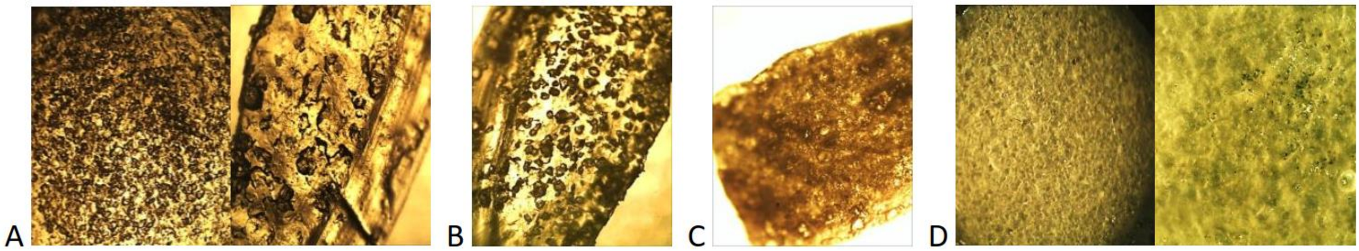

Figure S1. Freshly prepared (**A**); 5 min (**B**); 1 day (**C**) and 1 week (**D**) conditioned membranes containing TBATBB and dry KCl. Conditioning solution: 0.01 M KCl.

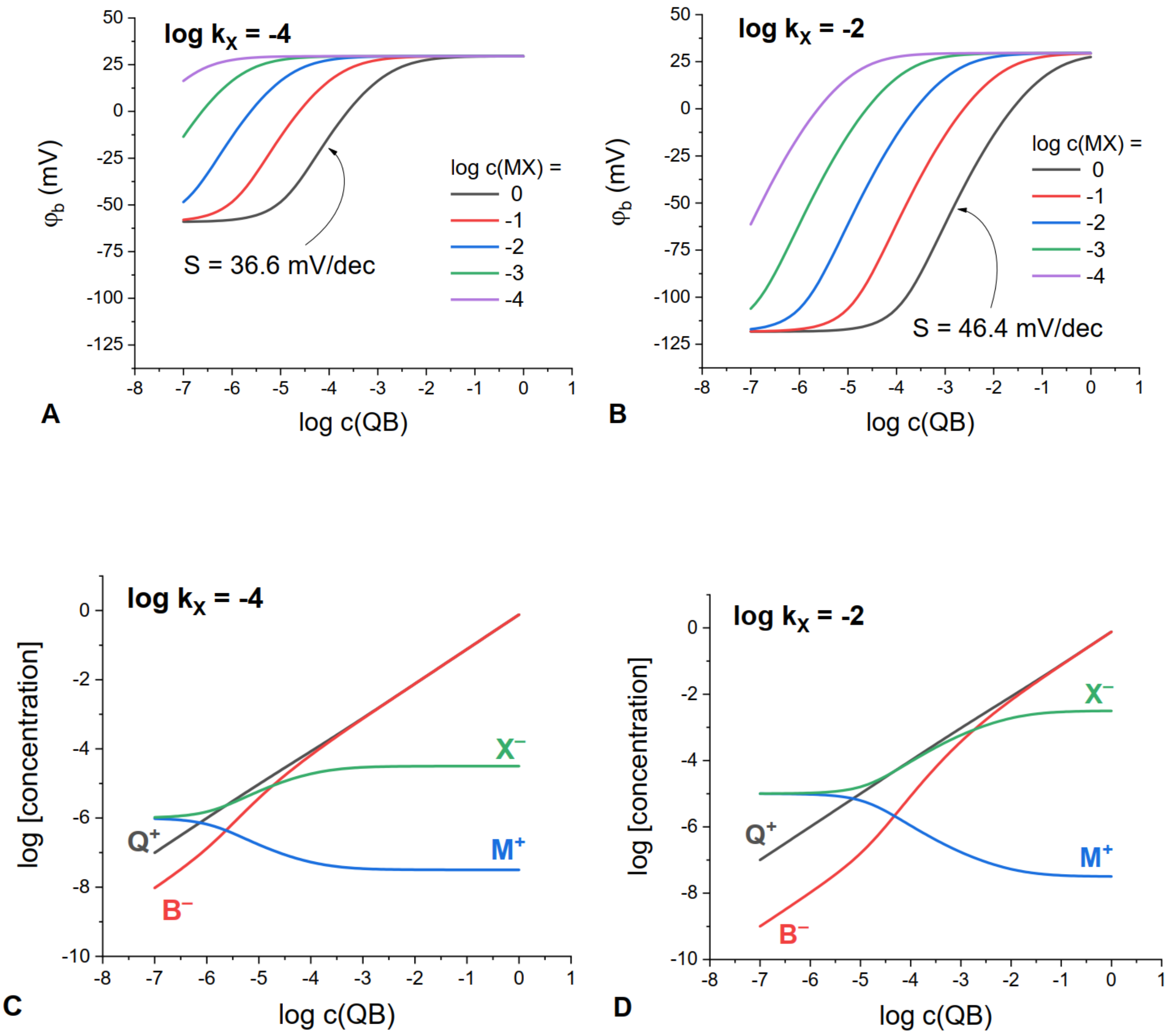

Figure S2. (**A**, **B**) Calculated dependences of the phase boundary potential on the Q^+B^- content in the polymeric phase at varied MX concentration in the aqueous phase (indicated in the plot) for two different partition coefficients of the aqueous anion X^-. (**C**, **D**) The respective concentration profiles in the polymeric phase at $c(MX) = 0.1$ M. Ionic partition coefficient of X^- is indicated in the plot. $k_M = 10^{-6}$; $k_Q = 10^3$, $k_B = 10^2$.

Table S1. The characteristics of the response of the electrodes containing both KT*p*ClPB and ETH500 in the membrane.

	KCl	NH4Cl	CsCl	NaCl
mean slope for Nernstian response (below equimolar Q^+B^- to IR ratio), mV/dec	57.5 ± 0.5	57.5 ± 0.6	57.4 ± 0.4	59.0 ± 0.5
mean slope (above equimolar Q^+B^- to IR ratio), mV/dec	2.5 ± 1.3	5.2 ± 1.4	0.8 ± 1.8	3.8 ± 0.8
mean E value, mV	99.4 ± 5.2	140.1 ± 8.8 mV	125.0 ± 4.8	155.7 ± 6.2

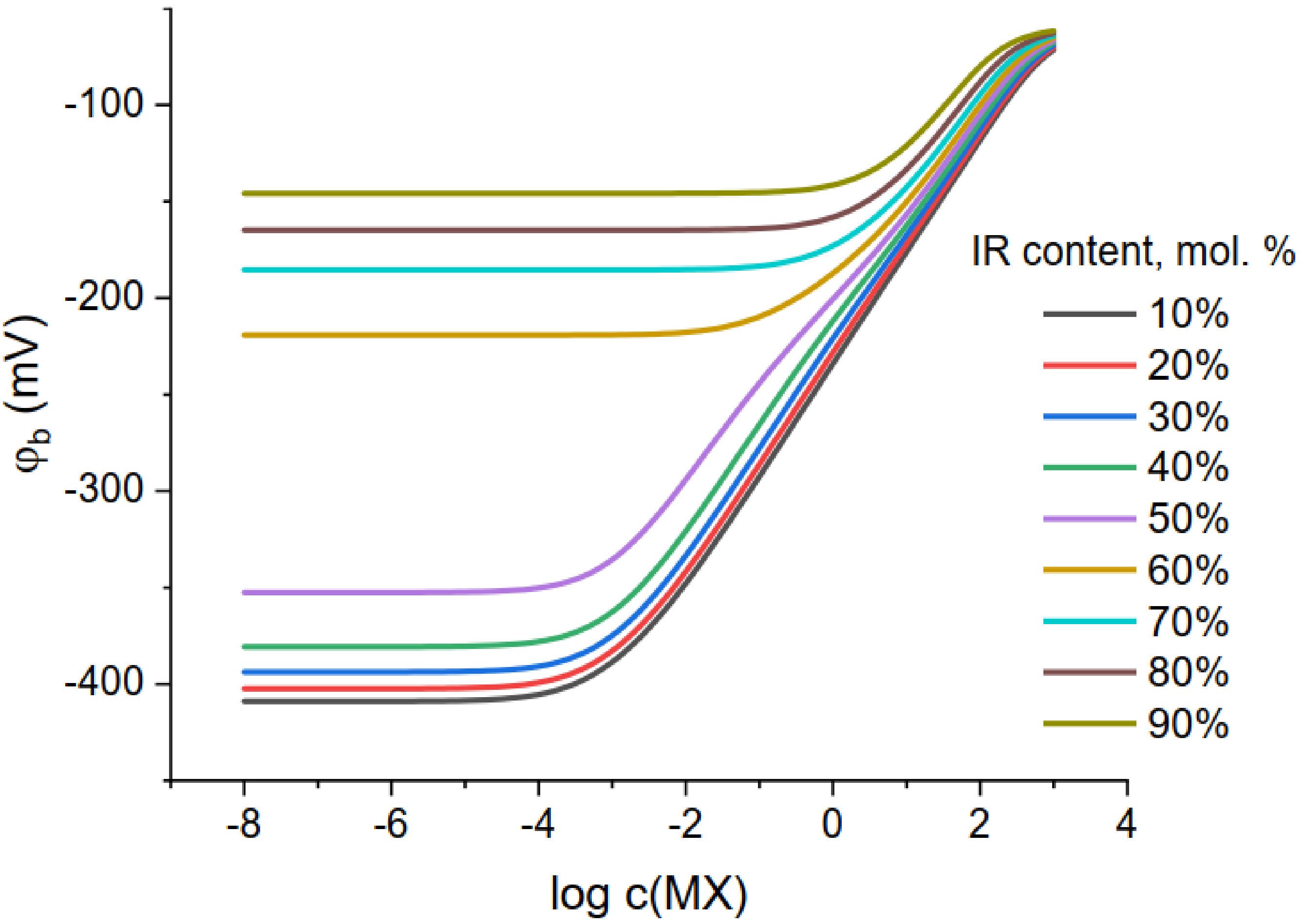

Figure S3. The simulated response curves for the membranes containing both IR and Q^+B^- in molar ratios from 10 to 90% (indicated in the plot). $k_M = 10^{-6}$, $k_X = 10^{-4}$; $k_Q = 10^9$, $k_B = 10^5$; $k_I = 10^{-5}$;$c(QB + IR) = 10^{-2}$ M.
